# Effectiveness of Teaching Mini Handball through Non-Linear Pedagogy in Different Socioeconomic Contexts: A Pilot Study

**DOI:** 10.3390/ijerph192013002

**Published:** 2022-10-11

**Authors:** Sebastián Espoz-Lazo, Claudio Farías-Valenzuela, Victor Reyes-Contreras, Paloma Ferrero-Hernández, Frano Giakoni-Ramírez, Mauricio Tapia-Zavala, Daniel Duclos-Bastías, Pedro Valdivia-Moral

**Affiliations:** 1 Facultad de Ciencias Para el Cuidado de la Salud, Universidad San Sebastián, Lota 2465, Providencia 7510157, Chile; 2 Instituto del Deporte, Universidad de Las Américas, Santiago 9170022, Chile; 3 Facultad de Educación y Ciencias Sociales, Universidad Central, Lord Cochrane 418, Santiago 8330546, Chile; 4 Facultad de Educación y Cultura, Universidad SEK, Santiago 7520317, Chile; 5 Faculty of Education and Social Sciences, Universidad Andres Bello, Santiago 7550000, Chile; 6 Departamento de Educación Física, Deportes y Recreación, Universidad Metropolitana de Ciencias de la Educación, Santiago 7520317, Chile; 7 Escuela de Educación Física, Pontificia Universidad Católica de Valparaíso, Valparaíso 2374631, Chile; 8 Facultad de Ciencias de la Educación, Universidad de Granada, 18071 Granada, Spain

**Keywords:** sport pedagogy, motor skills, team sport, scholars, pre-sports

## Abstract

Mini handball is among the sports included as part of school physical education in Chile to improve children’s motor skills and to motivate their adherence to a healthy and active lifestyle in response to concerns about this country’s high level of childhood obesity. To this end, non-linear pedagogy (NLP) has been used to develop motor skills through mini handball in the school context. However, socioeconomic differences that influence the development of children’s motor skills have not been considered to determine whether the methodology applies to everyone. The aim of the present observational study is to describe and compare the effectiveness of the previously applied NLP methodology in two contrasting socioeconomic contexts to determine whether it helps to develop motor skills through mini handball in both school contexts. The Levine test was used to determine the homogeneity of the variances (*p* < 0.05), as the distribution of the data was not normal. The Kruskal–Wallis H statistical test was used to analyse within-group data. Additionally, the Mann–Whitney U test was applied for comparisons between groups. The results show significant improvements in the acquisition of the expected motor skills specific to mini handball. Additionally, a shortening of the gap was evidenced between the groups during the training process, with no significant differences at the end of the progression. Therefore, the investigated NLP is equally as effective for schoolchildren in two opposite socioeconomic contexts.

## 1. Introduction

The rate of childhood obesity in Chile among the highest in the world [1,2]. In response to this concerning trend, the Chilean government has applied several plans at multiple levels to change people’s sedentary behaviours and nutritional habits [3]. 

One such plan was to modify the objective of physical education to reorient teaching practice from developing sport techniques in children to improve their sport performance so they can become future athletes [4] to the current aim, which is to develop children’s motor skills to eradicate sedentary behaviours, encouraging them to add physical activity to their daily interests [5].

The rationale for this political measure is based on the effectiveness of developing motor skills during childhood and its direct impact the level of adherence to physical activity and the acquisition of healthy habits throughout life [6]. 

In this sense, the Chilean physical education and health curriculum requires children to experience multiple sports and physical activities, such as team sports, corporal expression, and dance [7,8]. In particulars, in fifth and sixth grades, mini handball is among the team sports that must be taught effectively, as it involves all the basic motor patterns, as well as basic motor skills such, as running, jumping, catching, throwing, dribbling, pushing, unmarking, and motor decision making, among others, that every child must improve to achieve a healthy, active lifestyle [9,10].

In the last 5 years, various methodologies have been applied in different school contexts to teach handball, such as teaching games for understanding, integrative methodology, and the Handball at School program, with very positive results [11,12,13]. Non-linear pedagogy (NLP) has also been used as an alternative to develop motor skills through mini handball [14], as, according to this methodology, the human being is considered a biological system with constant interaction between all its component elements (physical, biochemical, emotional, etc.), as well as with the environment and peers, enabling navigation of situations through the exploration of actions that emerge from experience and skills relevant to the context in which they are developed [15]. 

NLP has been promoted as an effective and efficient methodology that allows children to explore movement skills based on key ideas and principles based on the teaching and learning processes contextualized in terms of the logic of dynamic interactions [16].

In terms of methodology, NLP differs from traditional models by equipping participants to solve motor challenges [15]. Coaches/teachers present a specific scenario in which participants have to explore alternatives using their motor skills and communication abilities or attempt to develop them to solve problems and overcome constraints relative to a goal [16] Feedback is later given to the participants by asking them questions to allow them to understand what they have done. 

Exploration is continuous and multimodal; it reveals information that is later used to perform actions. Exploration is encouraged and adapted to the context, the more alternatives are developed in response to challenges associated with changing scenarios and environments [17]. NLP invites children to explore as a result of the manipulation of constraints based on children’s ability to adapt and easily learn. Chow [16] showed how games can be redesigned so children can participate according to their current abilities in terms of age and physical maturation without sacrificing the key elements of the adult game, maintaining its representativeness [16].

In this sense, NLP has been used for mini handball in the school context, particularly for physical education, although with only one reported experience. Reyes-Contreras et al. [14] based their NLP proposal on Gibson’s ecological model [18]. The researchers designed and applied a methodological progression with evolving levels of complexity, obtaining favourable results in the acquisition and development of motor skills through mini handball involving multiple constraints [14]. However, the researchers did not report details about the socioeconomic background of the children who participated in the study, although it is well-known that when different socioeconomic strata are compared, differences are often observed in terms of the levels of development in both boys and girls between high- and low-income groups [19,20,21]. The lack of socioeconomic data limits the possibility of transferring the results with respect to the NLP methodology used to develop motor skills through mini handball to other environments.

It is important to develop an effective methodology that transcends socioeconomic differences to achieve the goal of improving every child’s health as set out in the Chilean National Survey of Physical Activity and Sports Habits 2021 For the Population Aged 5 and Over [22]. Children in lower-income social strata tend to be highly physically inactive compared to those in higher-income strata [23,24]. 

Therefore, the aim of this study is to describe and compare the effectiveness of the NLP methodology previously applied by Reyes-Contreras et al. [14] in two contrasting socioeconomic contexts to determine whether this particular methodology could be useful to develop motor skills through mini handball in different socioeconomic school contexts.

## 2. Materials and Methods

### 2.1. Design

This is an observational study with the aim of obtaining relevant information according to the objective of this research. In particular, this corresponds to an ideographic and multidimensional follow-up design, including an introduction and intersession analysis, through which information was obtained on the frequency of the motor skills performed by the participants [25].

### 2.2. Participants

A total of 22 schoolchildren (15 boys and 7 girls; BMI = 19.6 + 0.5; age, 10.7 + 0.6 years) in the fifth and sixth grades of primary Chilean school education were invited to voluntarily participate in the study. 

Among the 22 participants, 11 children attended a private school (G1) and were classified as Chilean socioeconomic type C1a, meeting the criteria of a high economic family income, low vulnerability, high level of access to goods and services, quality food, and positive use of leisure and recreation time [26].

The other 11 children attended a public school (G2) and were classified as Chilean socioeconomic type D, meeting the criteria a medium–low economic family income, a medium but unstable rate of vulnerability, their family core remaining indebted, concern associated with keeping their jobs, consumption of products according to price and not quality, and almost no leisure time as they find themselves with many hours of work and transfers [26].

For G1, their school curriculum prescribes eight hours of physical activity per week during the semester of classes, divided between physical education classes (two hours per week) and multisport workshops (six hours per week).

The G2 children participated in regular physical education classes during the semester, comprising only 2 h per week of planned physical activity at school.

Participants in both the G1 and G2 groups had no previous experience in mini handball.

### 2.3. Procedure

Before the investigation started, both the parents/guardians and the participants were notified and informed about the study procedures, and their participation was voluntary. 

This research was approved by the Research Ethics Committee of the University of Granada under registration number 2000/CEIH/2021 and conducted according to the standards of the Declaration of Helsinki [27], specifically regarding research and medical procedures involving children.

Both the G1 and G2 groups underwent 12 training sessions (2 weekly sessions), which included a 10 min warmup comprising neuromuscular activation exercises and multidirectional displacement execution actions, followed by the main activity as described the Reyes-Contreras et al. [14] involving the study of mini handball motor skills using NLP methodology. The training sessions concluded with cool-down exercises involving relaxation and stretching. 

### 2.4. Non-Linear Pedagogy Methodology

Guided by the aim of the present research, we applied the same methodology applied in the study by Reyes-Contreras et al. [14]. 

A limited space throughout the court was marked with 4 cones within which 4 participants had to perform as attackers during the exercise. One player was situated close to each cone to play the roles of receiver and passer. This exercise comprises 4 stages in which 4 defenders were included from stage 2 to stage 4. In each stage, defenders had fewer limitations on their actions, presenting more complexity for the attackers to solve the motor challenge. 

The main goal of the exercise was to solve technical–tactical tasks based on the management of the following constraints:

- Move exclusively through the areas delimited in the exercise within the game field;
- Accomplish 6 passes before completing the task objective; and
- Carry out the activity in less than 30 s at the maximum possible speed.

The goal was to stand in the centre of the marked zone with the handball ball before the time ends.

The protocol described in the aforementioned study and used in the present study, comprises following:

- Before the activity started, all three constraints were explained to each participant, along with the task instructions and the objective to be achieved;
- Participants were reminded about the mini handball regulations that directly affect the actions to be executed, i.e., the rules of the 3 steps and double dribbling;
- No further instructions were provided, except for the starting and ending signal.

As in the Reyes-Contreras et al. [14] study, every 3 sessions, the difficulty level was increased by manipulating another variable [28], i.e., the inclusion of a defender, whose function evolved from being passive to being intensively active [14]. 

### 2.5. Data Collection

For data collection, all sessions were recorded with two Sony^®^ Handycam DCR-SX22 cameras located at approximately 4 ms height [29] to cover the entire execution field, avoiding blockages toward the view of the camera between the participants. 

All recorded sessions were saved in a virtual hard drive for later editing to highlight every time the exercise started and finished. All edits and retrospective observations were performed using Kinovea^®^ software and Windows Media Player^®^, respectively. 

For observational analysis, an ad hoc observation instrument described by Anguera and Mendo [28] and adapted by Reyes-Contreras et al. [14] was used. This instrument contemplates a system of non-exclusive exhaustive categories in which the independent variables are described to make the observation as specific as possible (Table 1) [30].

A group of three observers, all physical education teachers and handball coaches, were trained to analyse mini handball motor skills through observational methodology using the ad hoc instrument. A professional handball expert with experience in this type of investigation trained the observers.

To ensure correct data collection, observers met with a handball expert after their training to clarify any doubts they may have had about the observation process. A pilot video of a previous experiment was provided to the observers to practice the observational methodology, with similar results obtained for all three observers.

Soon after, all videos were randomly organized into three groups to later be distributed among the observers for evaluation.

All observers had to identify each participant’s emergent mini handball motor skill during the NLP exercise in each session and later qualify it as effectively performed (EP) every time an observed player achieved a successful mini handball motor skill or non-effectively performed (NEP) every time an observed player intended to perform a motor skill without success as previously described. 

All results were arranged in an Excel^®^ spreadsheet using a numeric code for each qualification (EP = 1; NEP = 0) for which a list of correctly and incorrectly performed handball motor skills were noted.

### 2.6. Statistical Analysis

IBM SPSS Statistic Software^®^ v25.0 (SPSS Inc., Chicago, IL, USA) was used for data analysis. Descriptive statistics are reported as means and standard deviations. 

The Kolmogorov–Smirnov test was applied to check the normality of the variables because the number of observations was greater than 50.

As the distribution of the data was not normal, the Levine test was applied to check the homogeneity of the variances with a *p*-value of <0.05 for all variables. The non-parametric Kruskal–Wallis H statistical test was used to independently analyse the data obtained at 4 different moments for the observed actions of the G1 and G2 groups.

In parallel, to analyse the differences between the groups, the Mann–Whitney U test was applied to compare independent samples.

## 3. Results

The total number of observations made during the data analysis for each constraint was considered for descriptive statistics, as well as the total number of times each motor skill was effectively performed (Table 2).

**Figure 1:**
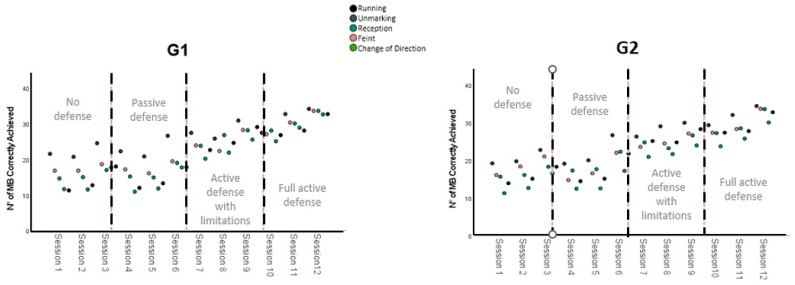
Evolution of the variables of G1 and G2: running, unmarked, reception, feint, and change of direction 4.

**Figure 2:**
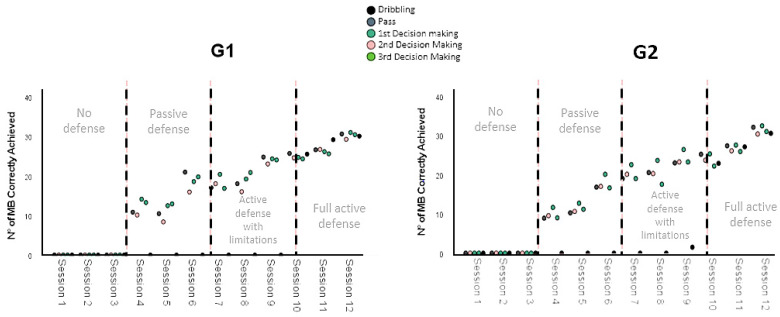
Evolution of the variables of G1 and G2: dribbling, pass, first decision making, second decision making, and third decision making.

The results of the Kruskal–Wallis H test showed an asymptotic sigma of *p* < 0.005. These data indicate that for the actions of the G1 and G2 groups, during the evolution of the training process, there were modifications in the amount of emergent mini handball motor skills. To determine whether these modifications were negative or positive, the data were displayed in a graph of grouped dispersion points for different variables (Figure 1 and Figure 2).

The graphs show an upward trend with respect to the number of emergent motor skills detected for each group during the progression of sessions in the training process. Therefore, as the training process advances and the complexity of the motor scenario is modified, the subjects in both groups demonstrate an improvement in the effective execution of the handball motor skills.

The results of the Mann–Whitney U test for two independent samples (Table 3) indicate that at the beginning of the training process with the NLP methodology, no significant differences were observed between the G1 and G2 groups in terms of the execution of the emergent motor skills, except for passes.

At the end of the first constraints, there were significant differences in terms of the number of successful motor skills executed related to the approach run through the delimited area in the exercises (running) and passes between attackers and receivers (passes) but not for any of the other motor skills observed. A similar phenomenon occurred later when comparing the results of the two groups with increasing level of complexity of the motor scenario. The data indicate that after completing the exercise with constraint 2, there were significant differences in terms of the application of motor skills, particularly in driving the ball through the field of action (dribbling) and passing (pass), as well as in the second decision-making task (second decision making). However, for the third constraint, the results were similar between the two group, with significant differences only observed between the groups in terms of tactical behaviour of changes of direction. Finally, for the last modification (constraint 4), after 12 sessions, there was no significant difference between the G1 and G2 groups in terms of the execution of motor skills within the context of the game and the proposed training process.

## 4. Discussion

The fundamental objective of the present work was to describe and compare the effectiveness of a mini handball teaching method based on NLP as previously presented by Reyes-Contreras et al. [14], in two socioeconomically opposed educational contexts.

The results show that for both intervened groups, the use of the NLP methodology resulted in an increase in the number of effective executions of motor skills comparing the evolution of the process in both G1 and G2 independently. This phenomenon is explained by the fact that each phase of the applied methodology implied the repetition of three training sessions with the same characteristics and that in each session, the same type of constraints had to be repeated under the same structure.

According to Kee [31], the development of motor skills occurs under a three-stage scheme: (1) cognitive stage: the subjects make conscious efforts to remember the instructions during the desired execution of the motor skills; (2) associative stage: the subjects rely less on the provided instructions and familiarize themselves with execution through experience, strengthening their motor response based on the situational demands; and (3) autonomic stage: the subjects internalize the ability, allowing them to execute actions automatically with less use of cognitive analysis. The three sessions that make up each phase of the methodology applied in the present study foster an environment of repetitions of mini handball actions that can be processed under the three described stages.

Based on the aforementioned arguments, the decrease in errors and the ability to execute a greater number of motor actions in each of the phases of the applied methodology can support the progress of the subjects in the stages of learning motor skills. The beginning of each phase develops through the comprehension stage, whereas the end of each phase is executed within the automation stage. Similar results were reported in a study on mini handball by Camacho-Cardenosa et al. [32], in which the acquisition of motor skills related to precision and speed during ball manipulation were progressively improved with actions such as throwing runs and dribbling, until a significantly higher level was achieved.

In both the study by Camacho-Cardenosa et al. [32] and the present study, the tasks to which the participants were submitted were delivered progressively according to the level of difficulty. This might have supported motor development and adaptation, independent of the socioeconomic level of the participants, as when participants are exposed to conditions to which they are already accustomed, stability in behaviour is usually observed without significant alterations [33]. Initially, we expected that the G1 group would respond better than the G2 group due to their higher volume of weekly physical activity outside of the study. However, as noted in the results, both the G1 and G2 may have had sufficient motor experience so that the initial difficulty did not require adaptation.

During the progression of the methodology, when advancing to the second phase, we observed significant differences for some specific motor behaviours, favouring the G1 group over G2. This could be explained by the arguments of Suárez-Lopez and González Ardila [33], who points out that motor development depends on the time of practice, with formal and informal contexts being equally important for the acquisition of motor skills in children between the ages of 7 and 12 years. In this case, the G1 group presented higher volumes of complementary physical activity relative to the G2 group, which may have promoted the initial development of motor skills.

The same phenomenon is explained by Venetsanou and Kambas [34], who describe that in most of investigations referring to the development and learning of middle-class or lower-class children, difficulties are encountered in the learning processes due to feeding problems in the lowest and most vulnerable socioeconomic strata but also due to the limits imposed by parents who cannot encourage their children to practice sports. This was later reaffirmed by Cano [35], who explained that children from working-class families have worse cognitive outcomes and more behavioural problems than children from upper-class families.

In addition, a contemporary study concluded that child development in all dimensions is strongly dependent on the socioeconomic stratum to which individuals belong, as children from lower strata present with a series of affective, spatial, economic, and social deficiencies [36]. However, the applied non-linear pedagogy training process seems to mitigate these differences, resulting in motor learning with similar values in terms of effective achievement of specific mini handball motor actions at the end of the entire process in both groups.

Regarding the difference between the volume of complementary physical activity, this does not necessarily result in a greater number of repetitions of specific mini handball motor actions; however, this difference may have been associated with more general motor experience in the G1 group, which could explain the initial observed difference between the two groups. However, the proposed non-linear pedagogy method balances this difference by allowing “noise” to be part of the development of the acquisition of expected motor behaviours [37]. In the same vein, in a study by Gómez-Criado and Valverde-Esteve [38], in which non-linear pedagogy was applied to the volleyball unit of a physical education class in a high school, a similar improvement was observed to that reported in our study; the developed motor behaviour improved in quality, accompanied by greater and better decision-making by the participants during the game actions. In this study, the methodology was replaced in the regular course of physical education, so there was no increase in the volume of extracurricular motor experience. Even so, significant improvements were reported compared with those who were not subjected to this methodology.

A limitation of the present study is that although several studies have been conducted related to mini sports, few studies have investigated the improvement of specific teaching processes for mini handball as a basis for the training of athletes; likewise, no other studies have been conducted on the use of non-linear pedagogy in this context. Therefore, it was not possible to conduct a more in-depth analysis in and to compare our with those of previous studies to enrich the discussion.

Finally, the results obtained in this study are consistent those of previous studies in which non-linear pedagogy was applied practically and experimentally [35,36,37,38,39,40,41]. The use of constraint manipulations, whereby actions are limited, and objectives are forced, promoted the development of motor behaviours and tactical decisions of the participating subjects during each exercise despite not having requested them, indicating the effectiveness of the method in achieving the motor goal.

## 5. Conclusions

The findings of this research regarding the effectiveness of the use of an applied methodology for the development of motor skills in mini handball based on non-linear pedagogy indicate that the proposed methodology may be effective despite the existing gaps in the process of learning the motor skills of mini handball, regardless of previous motor experience of the participants. At the end of the process, most of the individuals achieved the same expected behaviours. Further investigations involving experimental and control groups are required to confirm these results.

Furthermore, the methodology of non-linear pedagogy, seems to be an effective tool to improve and develop mini handball skills in a short period. Therefore, non-linear pedagogy could be an effective methodology to be used in the school context as a strategy to improve adherence to a physically active lifestyle.

## Figures and Tables

**Table 1:** Mini handball motor skills.

Behaviours	Description
Running	Moving on speed to look for the appropriate distance to comfortably and effectively receive a pass without opposition
Unmarked	Move away from the defending player to a favourable receiving location by running
Reception	Secure control of the ball following a pass from another player, receiving the ball comfortably with fluid movements
Feint	Execution of a feint to condition the behaviour of the opposing defender after receiving the pass by the attacking player
Dribbling	Driving the ball through the game field, manipulating it by hand, throwing it toward the ground and, and receiving it again according to the characteristics described in the handball regulations, avoiding loss of control due to disturbances by the defender in the lines of movement of the attacker
Change of direction	Explosive change of direction, causing the attacker to pass the defender, leaving him free on the path to the objective
First decision making	Search for a free receiver and for an advantageous position to achieve the objective, using the cycle of steps allowed by the handball regulations to deliver a pass to the receiver
Second decision making	Search for and use free space, using the cycle of steps allowed by the handball regulations, achieving the use of the space in advance of another participant during the exercise
Third decision making	Search for a passer in a position of advantage for free reception after recovering the ball
Pass	Deliver the ball to a receiver at the appropriate speed and force so that the receiver receives it comfortably and safely

Adapted from [14].

**Table 2:** Descriptive statistics.

	Group 1								Group 2								Total of Observations
Motor Skill	Const. 1	SD	Const. 2	SD	Const. 3	SD	Const. 4	SD	Const. 1	SD	Const. 2	SD	Const. 3	SD	Const. 4	SD	
Running	22.3	2.6	23.2	3.5	28.1	3.1	31.5	4.0	20.5	3.2	21.9	4.8	28.6	3.7	31.7	3.9	264.0
Unmarking	0.0	0.0	14.1	5.2	19.9	4.6	27.6	6.2	0.0	0.0	12.0	5.2	21.1	5.9	27.9	6.4	264.0
Reception	17.4	2.0	17.6	2.3	24.8	3.6	30.4	4.3	18.5	4.0	17.8	5.2	25.2	3.1	29.6	4.0	264.0
Feint	0.0	0.0	11.5	3.3	19.0	5.2	26.8	6.5	0.0	0.0	12.4	4.5	21.3	3.5	26.6	5.4	264.0
Dribbling	15.5	1.5	16.4	2.8	26.3	4.3	30.6	3.0	16.7	4.3	19.1	4.7	24.9	5.0	29.6	4.0	264.0
Change of direction	0.0	0.0	15.0	2.9	21.3	4.1	27.2	5.4	0.0	0.0	14.8	4.9	24.2	3.6	28.4	4.3	264.0
First decision making	13.6	3.4	13.5	3.4	22.5	3.8	28.9	5.4	13.5	4.2	14.1	3.4	22.3	2.9	26.4	4.8	264.0
Second decision making	0.0	0.0	15.3	3.2	20.5	4.2	26.7	6.0	0.0	0.0	12.3	4.6	20.4	6.0	26.0	6.7	264.0
Third decision making	0.0	0.0	0.0	0.0	0.0	0.0	28.2	4.4	0.0	0.0	0.0	0.0	0.0	0.0	26.7	4.4	264.0
Pass	14.0	4.2	14.4	4.3	25.5	4.8	29.2	3.3	15.8	4.1	17.1	6.3	26.3	4.5	29.0	4.5	264.0

Const. = constraint; Const. 1: no defence; Const. 2: passive defence; Const. 3: active defence with limitations; Const. 4: full active defence; SD = standard deviation.

**Table 3:** Asymptotic sigma values of the Mann–Whitney U test for comparison between groups G1 and G2.

	Pre	Const. 1	Const. 2	Const. 3	Const. 4
Running	0.103	0.016 *	0.218	0.342	0.948
Unmarked	1.000	1.000	0.313	0.410	0.867
Reception	0.320	0.536	0.974	0.780	0.337
Feint	1.000	1.000	0.356	0.067	0.580
Dribbling	0.477	0.958	0.009 *	0.296	0.367
Change of directions	1.000	1.000	0.338	0.004 *	0.458
First decision making	0.586	0.821	0.301	0.959	0.053
Second decision making	1.000	1.000	0.002 *	0.959	0.642
Third decision making	1.000	1.000	1.000	1.000	0.080
Pass	0.039 *	0.067 *	0.029 *	0.752	0.923

* Significant differences *p*-value = “asymptotic sigma” < 0.05; Const. = constraint; Const. 1: no defence; Const. 2: passive defence; Const. 3: active defence with limitations; Const. 4: full active defence.
